# Genetic Parameter Estimates for Growth, Meat Yield and Foot Color Traits of Pacific Abalone *Haliotis discus hannai*

**DOI:** 10.3390/ani16050782

**Published:** 2026-03-02

**Authors:** Shoudu Zhang, Tianyi Xu, Ming Li, Longwei Dai, Zhenlin Hao, Fucun Wu

**Affiliations:** 1 State Key Laboratory of Mariculture Breeding, Ningde 352108, China; shouduzhang@163.com; 2 Marine Science Research Institute of Shandong Province (National Oceanographic Center, Qingdao), Qingdao 266000, China; 3 School of Marine Sciences, Ningbo University, Ningbo 315211, China; 24tyxu@stu.edu.cn; 4 Key Laboratory of Marine Biotechnology of Guangdong Province, Shantou University, Shantou 515063, China; 5 Zoneco Group Co., Ltd., Dalian 116001, China; bioliming@163.com; 6 Key Laboratory of Mariculture and Stock Enhancement in North China’s Sea (Dalian Ocean University), Ministry of Agriculture, Dalian 116023, China; 18840870182@163.com (L.D.); haozhenlin@dlou.edu.cn (Z.H.); 7 Institute of Oceanology, Chinese Academy of Sciences, Qingdao 266000, China

**Keywords:** *Haliotis discus hannai*, foot color, meat yield, growth, heritability, genetic correlation

## Abstract

This study was designed to evaluate the genetic parameters of growth, meat yield and foot color in Pacific abalone (*Haliotis discus hannai*) to support the development of a quality-oriented breeding program. Using data from 141 pedigreed full-sib families, heritabilities and genetic correlations among these traits were estimated. The results indicated that shell length at varying ages (0.45–0.71) and total wet weight (0.48) exhibited moderate-to-high heritabilities, suggesting substantial potential for genetic improvement in growth traits. The heritabilities for foot color (0.26) and meat weight (0.31) were moderate and statistically significant (*p* < 0.05), whereas the heritability for meat yield (0.14) was low and not statistically significant (*p* > 0.05). Strong positive genetic correlations were observed between shell length and total wet weight at harvest (0.90), as well as between total wet weight and meat weight at harvest (0.92). In contrast, the genetic correlations between total wet weight and foot color, and between total wet weight and meat yield, were low and not statistically significant (*p* > 0.05). These findings suggest that direct selection can effectively improve the growth and meat weight of Pacific abalone. However, improving foot color and meat yield may require independent or multi-trait selection approaches. Overall, this study provides crucial data for formulating a comprehensive breeding strategy that integrates both production efficiency and product quality in Pacific abalone aquaculture.

## Simple Summary

This study performed a genetic evaluation of economically important traits in Pacific abalone to support quality-oriented selective breeding. Using 141 pedigreed families, we estimated heritabilities and genetic correlations for growth and processing-related traits. Growth traits, including shell length and total weight, exhibited moderate-to-high heritability, indicating substantial potential for genetic gain. Foot color and meat weight were moderately heritable, whereas meat yield showed low heritability. High genetic correlations were observed between growth traits and meat weight, but not between growth traits and foot color or meat yield. These results suggest growth and meat weight can be improved through direct selection, while enhancing foot color and meat yield may require trait-specific or alternative breeding strategies. Overall, this work provides a foundation for breeding programs aiming at balancing production efficiency and product quality.

## 1. Introduction

Abalones (family *Haliotidae*) represent a highly valued group of marine gastropod mollusks in global fisheries and aquaculture. While most wild abalone fisheries are facing over-exploited or depleted conditions, thereby limiting further expansion, aquaculture production has experienced rapid growth over the past three decades [1]. The abalone aquaculture industry in China, in particular, has undergone dramatic expansion, accounting for approximately 90% of global output with annual production ranging from 180,300 to 217,831 tons over the last five years [2,3,4]. *Haliotis discus hannai* Ino, commonly known as the Pacific abalone, is the predominant cultured species and a vital source of high-quality seafood in East Asia [4,5]. Since its introduction to southern China, *H. discus hannai* has become the dominant species in intensive coastal farming systems [3,6]. As annual production in China has reached a plateau, the industry’s strategic priority has shifted from quantitative expansion to qualitative genetic improvement and quality enhancement.

Traditional breeding methods, including selective breeding, crossbreeding, and polyploidy induction, have successfully enhanced growth and environmental stress tolerance in various abalone species [7,8,9,10,11,12]. In *H. discus hannai*, improved strains developed through selection, crossbreeding or hybridization are now commercially utilized in China [3,6]. More recently, advanced genomic tools such as high-density single-nucleotide polymorphism (SNP) arrays and genomic selection have emerged to accelerate genetic gain [13,14]. However, existing breeding programs predominantly target growth rate, survival, feed efficiency, and thermal tolerance [12,15]. Processing-related traits such as meat yield and meat/foot color have received comparatively less attention. As market competition intensifies and consumer preferences evolve, improving these quality traits is becoming increasingly important for sustaining economic returns and industrial competitiveness [3].

Meat yield and external appearance are critical economic traits in aquaculture, directly influencing processing efficiency and consumer acceptance [16,17]. Variations in meat yield and biochemical composition—including glycogen, protein, and collagen content—significantly affect nutritional value and texture [14,18]. Given that deep muscular foot color is generally undesirable for abalone processing, improving this trait is a primary commercial consideration. Meat and shell color also play a crucial role in market perception, especially for premium live or whole-shell markets [19]. The successful enhancement of product value through selective breeding for shell color and/or pigmentation in species like scallops and prawns [17,20] provides a precedent for similar efforts in abalone, where selection for favorable color traits is now becoming a breeding priority [8]. Accordingly, to support effective selection and to determine whether these traits can respond to breeding, estimating the genetic parameters for these traits in *H. discus hannai* is a prerequisite.

Recent advances in genetic evaluation research have substantiated the genetic improvement potential of numerous economic traits in abalone through selective breeding. Moderate-to-high heritabilities for shell length and body weight have been reported across multiple species, including *H. rufescens*, *H. laevigata*, *H. midae*, and *H. discus hannai* [15,21,22,23]. These findings validate the idea that conventional selection can deliver substantial genetic gains, as evidenced by multi-generation improvements in *H. rubra*, *H. laevigata* and *H. diversicolor* [8,24]. The scope of targeted traits in contemporary abalone breeding programs has expanded significantly in recent years. For *H. discus hannai*, Yu et al. (2022) demonstrated that residual feed intake (RFI) is heritable, with heritabilities ranging from 0.32 to 0.40, and is suitable for improving feed efficiency [15]. Liu et al. (2022, 2024) successfully applied the genomic prediction approach to improve heat tolerance in abalone *H. discus hannai* [4,13], while Winkler et al. (2024) first reported significant heritability for resistance against *Polydora hoplura* infestation [10]. In Australian abalone (*H. rubra* and *H. laevigata*), Kube et al. (2024) conducted a comprehensive analysis of large pedigreed datasets, finding moderate-to-high heritabilities for meat yield, confirming that processing and appearance traits possess exploitable genetic variation [8]. Although specific research on abalone flesh quality is limited, insights from studies on other mollusks provide valuable guidance. Research on Pacific oysters has mapped quantitative trait loci (QTLs) for glycogen and protein content [14,18], and the “Nan’ao Golden” scallop strain was successfully bred for high carotenoid content and shell color [17]. Notably, Liu et al. (2024) recently provided the first genetic parameter estimates for nutritional traits in *H. discus hannai* (glycogen, protein, and taurine), reporting moderate heritabilities (0.33–0.47) and demonstrating the feasibility of selection for quality [4]. Collectively, these studies indicate that a wide range of traits—from growth to nutritional composition—are amenable to genetic improvement.

Despite these advances, significant knowledge gaps persist regarding the genetic variations in processing traits in *H. discus hannai*. While meat yield has been investigated in Australian abalone species [8], systematic genetic evaluations for this trait in *H. discus hannai* remain scarce. Existing research has predominantly concentrated on shell size or total weight [15,23], largely overlooking the ratio of edible tissue to total body weight. Given that meat yield involves distinct resource allocation between somatic tissue and shell, it may not correlate strongly with simple growth metrics. Consequently, the absence of direct heritability estimates and genetic correlations complicates the design of effective selection indices for yield improvement. Furthermore, the genetic basis of shell/meat color or pigmentation in abalone is poorly characterized. Although certain genes involved in shell formation have been identified in mollusks [25,26], quantitative genetic analyses of color-related traits are limited. While heritability for foot color has been reported in hybrid Australian abalone [8], detailed genetic parameter estimates for meat and shell color in *H. discus hannai* are still lacking. Additionally, the relationship between phenotypic color expression and underlying biochemical pigment composition remains unexplored within a genetic context. Moreover, available genomic resources—such as whole genomes and SNP arrays—have not been fully leveraged to dissect the genetic architecture of processing-related traits. As a result, comprehensive data are lacking on the additive genetic variance of meat yield and color, their correlations with growth traits, and their expected response to commercial selection programs.

Current breeding objectives for the Chinese abalone industry continue to prioritize growth rate and survival, with limited emphasis on traits such as meat yield and coloration. However, neglecting these quality-related traits risks limiting future profitability in an increasingly differentiated market. Addressing these knowledge gaps is, therefore, essential. The present study aims to evaluate the genetic parameters for meat yield, meat color, and growth-related traits in *H. discus hannai*. Utilizing a pedigreed population, we estimated the heritability of these traits to assess their potential for direct genetic improvement. The results will provide foundational data for designing integrated breeding programs that enhance product quality. By addressing critical gaps in the literature, this work supports a strategic shift in abalone aquaculture—from a quantity-oriented model toward a balanced approach that prioritizes yield, quality, and resilience.

## 2. Materials and Methods

### 2.1. Population

The base population was established in 2019 using progeny from the Pacific abalone *Haliotis discus hannai* variety “Dalian NO. 1”. This variety, developed by crossing a Dalian, China stock (female parents), and an Iwate, Japan stock (male parents), was approved by the Ministry of Agriculture of the People’s Republic of China in 2004. Previous studies have shown that inter-population hybridization in this cross produces F_1_ hybrids with enhanced thermal tolerance, including improved survival and growth under elevated temperatures [3,5]. This documented heterosis for heat adaptation provided a strong biological rationale for deploying the “Dalian No. 1” variety in southern China. Accordingly, “Dalian No. 1” has been introduced from northern to southern China since 2006 and has been intensively cultured there for more than a decade. Full-sib and half-sib families were generated through a nested mating design, in which each sire was mated with two dams. A total of 141 families, resulting from matings between 81 sires and 124 dams, were obtained in the base generation.

### 2.2. Animal Rearing

Spawning and nursery activities were conducted at a commercial farm in Huilai County, Jieyang, Guangdong Province, China (116.45° E, 22.95° N), while the grow-out phase took place at a separate facility in Dongshan County, Zhangzhou, Fujian Province, China (117.43° E, 23.60° N). Fertilization was carried out in October 2020. Before spawning, putative broodstock were maintained in a conditioning system and individually marked with unique four-digit identification codes. On the evenings of spawning, broodstock were transferred into separate incubators constructed from foamed polypropylene, and spawning was induced by a combination of ultraviolet irradiation and controlled temperature shifts. Single-pair fertilizations were performed following the nested mating scheme described in Section 2.1. Abalone full-sib families were produced across two consecutive-day batches, and the resulting batch effect was considered negligible.

Juveniles reached a suitable size for physical tagging approximately 6 months post-fertilization [23]. At the time of tagging, the abalone had a mean body weight of 1.8 g with a standard deviation of 0.8 g. Each individual was tagged with a unique four-digit code affixed to its external shell surface. Approximately 80 offspring were tagged per full-sib family, resulting in a total of 12,800 tagged animals.

Following tagging, the abalone were reared under combined land-based and sea-based culture systems. During the warmer period (May to November), grow-out occurred in land-based flow-through tanks. From December to April, animals were transferred to a sea-based facility utilizing floating raft culture, consistent with established general husbandry approaches [3,6]. In the floating raft system, abalones were stocked directly into net cages suspended from rafts at the sea surface. Throughout the culture period, mean monthly water temperature varied from approximately 13 °C in February to about 28 °C in September. Routine husbandry, including tank or cage cleaning and feed management, adhered to standard commercial farm practices.

### 2.3. Data Collection

Measurements were taken from all surviving individuals at the final harvest in March 2023. The recorded traits included four shell-length measurements, total wet weight, soft-tissue weight, meat weight, meat yield, foot color, and survival. Phenotypic records for shell length at different growth stages were obtained by examining shell color marks, which were generated by changes in shell pigmentation due to alterations in feed type [23]. This method provided four shell-length measurements corresponding to ages 6, 18, 24, and 30 months for each surviving abalone at harvest.

**Figure 1:**
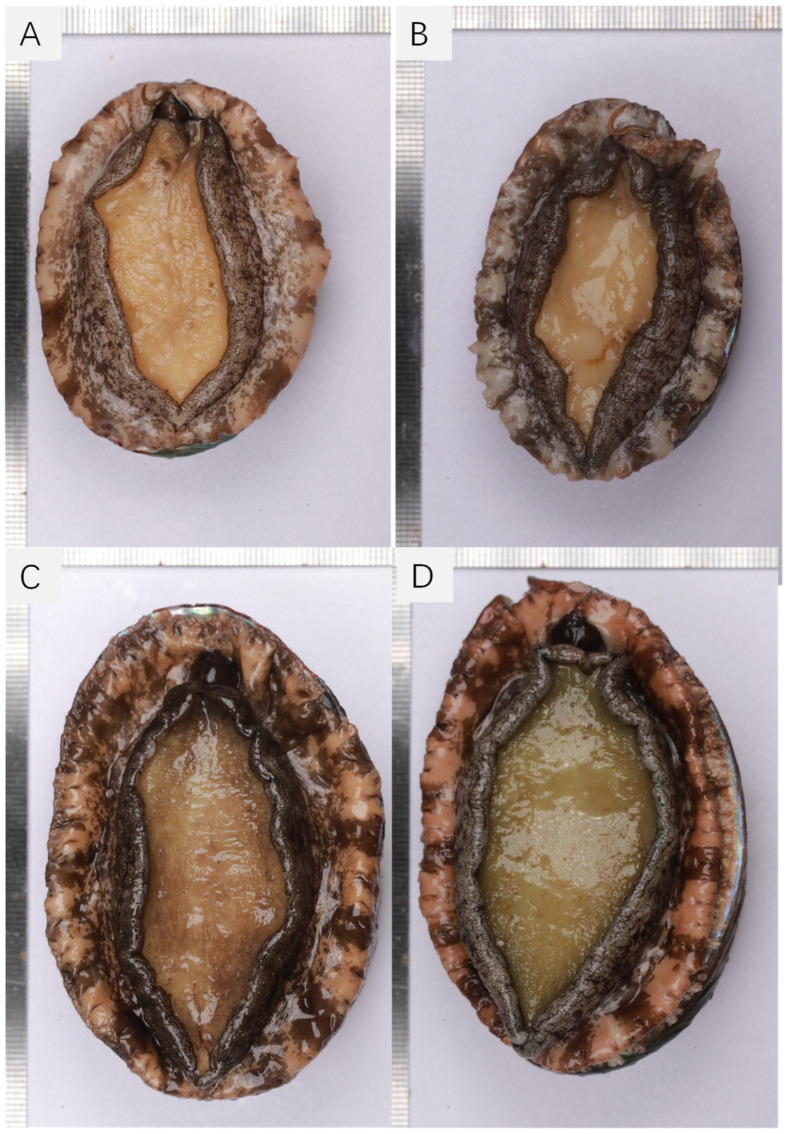
Representative phenotypes of foot color in Pacific abalone (*Haliotis discus hannai*). (**A**) Score 1: light color (pale yellow or cream) with little to no dark pigmentation; (**B**) score 2: light color, showing a mixture of light and dark patches; (**C**) score 3: intermediate color, characterized by black or dark gray pigmentation; (**D**) score 4: dark green color, with or without black or dark gray pigmentation.

Foot color was visually evaluated as a subjective indicator of pigmentation intensity on the sole of the foot. Scores were assigned on a four-point ordinal scale representing relative differences within the species, where a score of 1 corresponded to a pale-yellow foot (commercially preferred) and a score of 4 to a dark green to orange foot (least preferred). Representative images of acceptable and undesirable foot color phenotypes are provided in Figure 1.

While growth and foot color data were collected for the entire surviving population (n = 8001) using non-lethal methods, measuring meat traits required destructive sampling. Due to the economic cost of sacrificing potential broodstock and the labor intensity of dissection, we employed a random stratified sampling method to select 20–25 individuals from 30 full-sib families, resulting in a total of 680 individuals at 30 months of age for meat trait analysis. This subset was designed to capture within-family variation while preserving the majority of the population for future breeding. Soft-tissue weight was defined as the wet weight of the animal after shell removal, whereas meat weight was the wet weight of the muscle after removing both shell and viscera. Meat yield was calculated as the proportion of muscle weight relative to total wet weight, measured on a wet weight basis after removing shell and viscera.

Survival was calculated as the percentage of tagged abalones that remained alive at harvest, specifically as the ratio of the number of surviving individuals to the total number originally tagged.

### 2.4. Data Analysis

For univariate analysis of the foot color trait, we applied both a threshold sire–dam model (to estimate latent heritability) and a linear animal model. However, for the multivariate analyses involving growth and meat traits, foot color was treated as a continuous variable in a linear animal model. This approach was chosen because multivariate threshold–linear models frequently encounter convergence difficulties with complex pedigree data. Furthermore, linear models are known to provide robust estimates of genetic correlations for categorical traits when the number of categories is sufficient (i.e., >3) and the frequency distribution is not extremely skewed [27]. Variance components were obtained by restricted maximum likelihood and used to derive heritability estimates.

Specifically, an ordinal multinomial (threshold/probit) sire–dam model was employed for univariate analysis. The model was fitted using a threshold (liability) formulation. Specifically, an unobserved continuous latent variable, $l_{i}$ (liability), was assumed to underlie the observed ordinal phenotype:

$$l_{i}\;\;=\;m+G\;\;+\;sire+dam\;+\;f+e,$$

where ***G*** represents the fixed effect of gender, ***sire*** and ***Dam*** denote the additive genetic effects of the sire and dam, ***f*** is the common effect attributed to full-sibs, and e is the residual on the latent scale. A probit link was assumed by fixing the residual distribution on the latent scale to standard normal:

***e*** ∼ ***N***(0, 1),

Constraint Var(e) = 1 ensures the identifiability of the threshold model under the probit link.

For a probit multinomial sire–dam model with residual variance fixed to 1, the latent-scale heritability is calculated as

$$h^{2}={(\sigma }_{s}^{2}+\sigma_{d}^{2})/{(\sigma }_{s}^{2}+\sigma_{d}^{2}+\sigma_{f}^{2}+1)$$

The estimated common effect of full-sib families is calculated as

$$c^{2}\;=\sigma_{f}^{2}/(\sigma_{s}^{2}+\sigma_{d}^{2}+\sigma_{f}^{2}+1)$$

where $\sigma_{s}^{2}$ and $\sigma_{d}^{2}$ are the sire and dam random-effect variances, while $\sigma_{f}^{2}$ is the variance of the common effect for full-sibs.

Multivariate linear mixed models were applied to estimate genetic parameters for multiple traits analyzed concurrently. In total, ten traits were considered in this part of the analysis. For genetic analyses of the foot color trait, the ordinal score was treated as a continuous trait.

The general model fitted was:

***y*** = ***m*** + ***G*** + ***a*** + ***f*** + ***e***,

where ***y*** is the vector of observations for the trait(s) of interest, m is the overall mean, ***G*** denotes the fixed effect of gender, ***a*** is the random additive genetic (animal) effect, ***f*** is the random full-sib family effect, and ***e*** is the random residual. Random effects were assumed to be mutually independent and normally distributed with zero mean, with

$$\mathrm{Var}(a)=A\sigma_{a}^{2},\mathrm{Var}(e)\;=I\sigma_{e}^{2},$$

where ***A*** is the numerator relationship matrix derived from the pedigree, and ***I*** is an identity matrix.

Multivariate model structures were utilized. However, including all ten traits in a single multivariate model was not feasible due to issues with model convergence identified during preliminary analysis. Therefore, the first model included shell length at 6, 18, 24 and 30 months, along with total wet weight. Three additional bivariate models were constructed, each comprising total wet weight paired with soft-tissue weight, meat weight, meat yield and foot color. Survival was analyzed separately using a univariate threshold model.

A common environmental effect at the full-sib family level was initially incorporated into the model. However, models including this term did not converge reliably, and preliminary univariate analyses indicated that the corresponding variance component was very small for growth-related traits and negligible for foot color and meat yield traits. Consequently, the full-sib common environmental effect was not retained in the final models.

Narrow-sense heritability (h^2^) was computed as

$$h^{2}=\sigma_{a}^{2}/(\sigma_{a}^{2}\;+\;\sigma_{e}^{2})$$

where $\sigma_{a}^{2}$ is the additive genetic variance and $\sigma_{e}^{2}$ is the residual variance. Variance components and genetic parameters were estimated using mixed models implemented in ASReml-R (version 4.0), based on residual maximum likelihood (REML) [28].

## 3. Results

### 3.1. Phenotypic Data

The total number of full-sib families was 141, originating from 81 unique sires and 124 unique dams (Table 1). Of the 12,800 juveniles initially tagged, 9006 individuals survived the 30-month culture period, corresponding to a survival rate of 79.84%. Among the survivors, 8001 individuals retained their identity tags (87.73% retention rate). In total, 4210 were classified as female, 2983 as male, and 898 as unidentifiable. In addition, sex was not deliberately balanced across families. Data for shell length (SL), total wet weight (Tww), and foot color (Fc) were recorded for all identified survivors and assigned to their respective full-sib families. Of note was the absence of abnormal phenotypes for the trait of foot color, and the typical variation in pigmentation is shown in Figure 1. This figure depicts the morphology of *H. discus hannai* and specifically illustrates the four categories of foot color used in our scoring system. Shell length was recorded at four growth stages using distinct shell banding patterns induced by dietary manipulation. Family sizes ranged from 54 to 182 individuals for growth and color traits. A subset of 680 individuals was dissected to assess meat weight (Mw) and meat yield (My) (Table 1).

### 3.2. Data Summary

**Figure 2:**
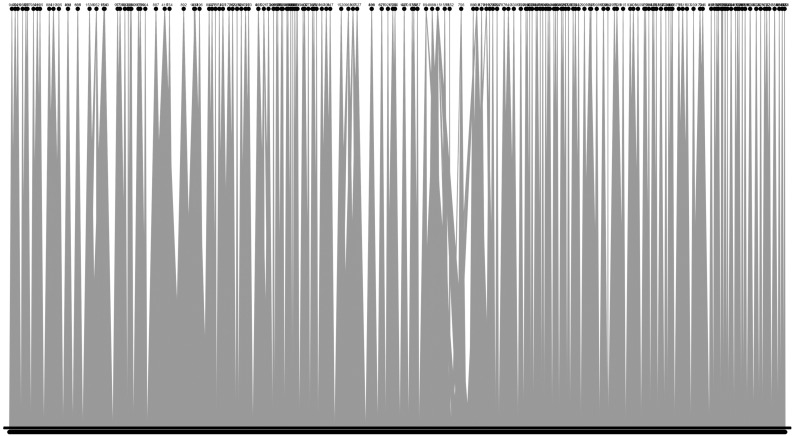
Pedigree structure of abalone samples in *H. discus hannai* population.

**Figure 3:**
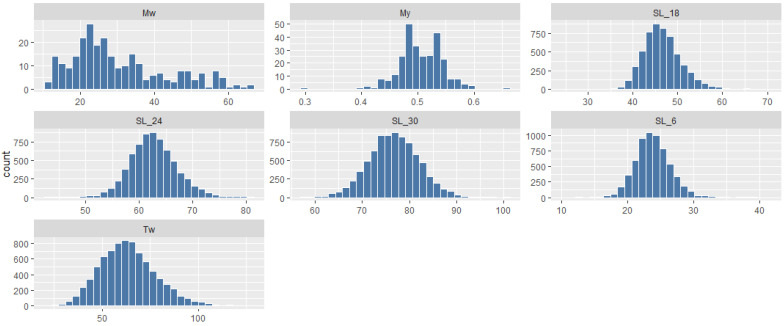
Phenotypic data distribution for each trait of *H. discus hannai* population.

**Figure 4:**
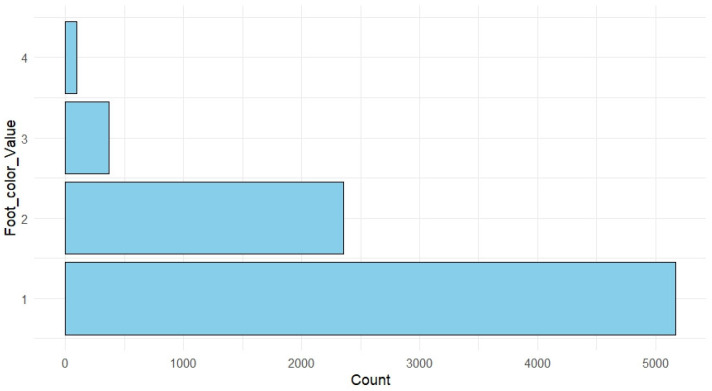
Frequency distribution (counts) of the four ordered phenotypic categories for abalone foot color (Fc). The x-axis shows the number of individuals, and the y-axis indicates the Fc category (1–4).

The pedigree structure is visualized using the R package + ggplot2 (version 3.5.2) in Figure 2. Each vertical line represents an individual, grouped by full-sib family, illustrating the overall family composition and the unbalanced contribution of individuals across families. Descriptive statistics for all the targeted traits are presented in Table 1. Mean shell lengths (±SD) at 6, 18, 24, and 30 months were 23.85 ± 2.68 mm, 46.53 ± 4.33 mm, 62.64 ± 4.46 mm, and 76.68 ± 5.34 mm, respectively. The mean total wet weight trait (Tww) at harvest was 63.36 ± 14.6 g, exhibiting substantial variation. Phenotypic coefficients of variation varied among traits but were notably higher for foot color, meat weight and meat yield. Most traits exhibited symmetric normal distributions as illustrated in Figure 3. Growth traits (shell length and total weight) exhibited a normal continuous distribution, suggesting their polygenic nature. In contrast, meat yield followed a normal distribution but with a slightly tighter range (Figure 3), reflecting the biological constraints of body proportion. The foot color scores showed a distribution where score 1 was the most frequent, flanked by fewer individuals in the dark (scores 3 and 4) categories (Figure 4).

### 3.3. Estimation of Genetic Parameters

Heritability estimates for shell lengths at different growth stages (SL_6, SL_18, SL_24, and SL_30) and total wet weight (Tww) at harvest are presented in Table 2. Heritabilities for shell length were moderate to high, ranging from 0.45 to 0.71, and exhibited a decreasing trend over time. The heritability of Tww at 30 months of age (0.48) was comparable to that of SL at the same age. All heritability estimates for growth traits were statistically significant (*p* < 0.05). Genetic and phenotypic correlations estimated using the multivariate animal model are also summarized in Table 2. Most genetic correlations were moderate (< 0.80), with the exception of the strong correlation between SL and TWW at 30 months (~0.90). All estimated correlations were statistically significant (*p* < 0.05).

Using the linear animal model, the heritability (± SE) for foot color (Fc) was estimated at 0.26 ± 0.03. In contrast, the cumulative (ordinal) multinomial threshold model yielded a higher latent heritability of 0.46 ± 0.06. Due to the lack of a direct formula for converting latent-scale parameters to the observed scale for multivariate ordinal traits, the observed heritability for Fc was not derived from the threshold model in this study.

Heritabilities for Tww, Fc, Mw, and My estimated by bivariate animal models were presented in Table 3. Heritability estimates for Fc (0.26) and Mw (0.31) were moderate and statistically significant (*p* < 0.05). In contrast, the heritability for My was low (0.14) and not statistically significant (*p* > 0.05). Genetic and phenotypic correlations between TWW, Fc, Mw, and My are also shown in Table 3. A strong genetic correlation was observed between TWW and Mw (0.92). However, genetic correlations between Tww and Fc, and between Tww and My, were very low and non-significant (*p* > 0.05).

## 4. Discussion

This study aimed to address a critical gap in the genetic evaluation of Pacific abalone (*Haliotis discus hannai*) by assessing processing- and quality-related traits (meat yield and foot color), alongside traditional growth traits. The primary objective was to generate foundational data for quality-oriented breeding programs in China by using a pedigreed culture population derived from the “Dalian NO. 1” variety. We observed moderate-to-high levels of heritability for shell length (0.45–0.71) and total wet weight (0.48), indicating substantial potential for genetic improvement in growth. Foot color (0.26) and meat weight (0.31) showed moderate and statistically significant heritabilities, whereas meat yield (0.14) had low, non-significant heritability. Strong positive genetic correlations were observed between shell length and total wet weight (0.90) and between total wet weight and meat weight (0.92). In contrast, genetic correlations of total wet weight with foot color and with meat yield were very low and non-significant.

The heritability estimates for shell length (0.45–0.71) and total wet weight (0.48) are consistent with previous reports of moderate-to-high heritabilities (0.2–0.6) in abalone [8,21,23,29], supporting the feasibility of selective breeding for increased size and weight. For instance, recent work on the South African abalone (*H. midae*) reported similarly moderate heritability for shell length (0.38) [29], and studies on the small abalone (*H. diversicolor*) have shown weight heritabilities ranging from 0.20 to 0.45 depending on the culture environment [24]. These consistent findings across species confirm that mass selection for growth rate is a viable strategy for abalone breeding globally. By contrast, the low and non-significant heritability for meat yield observed here suggests that direct selection for meat yield in *H. discus hannai* might be less efficient than for growth traits. The moderate heritabilities for foot color (0.26) and meat weight (0.31) are encouraging, indicating that these quality-related traits can be improved through selective breeding, which aligns with market demand for product quality.

In the estimation of genetic parameters, we employed an animal multivariate model that did not explicitly include a common environmental effect (c^2^) for full-sib families because of convergence issues. Although the nested mating design theoretically allows for the partitioning of c^2^ effects (such as separate tank rearing during the larval and nursery stages), our preliminary univariate analyses indicated that the variance attributable to the common environment was small for growth-related traits and negligible for foot color and meat yield traits. This suggests that the early transfer of juveniles to the standardized sea-based cage culture system minimized systematic environmental differences between families. Nevertheless, we acknowledge that completely omitting the c^2^ effect carries the risk of inflating additive genetic variance, as family-specific environmental advantages may be misattributed to genetics. This potential bias is generally most pronounced in early-stage traits (e.g., juvenile shell length) before individuals are acclimated to the common environment. Therefore, while our moderate-to-high heritability estimates indicate strong selection potential, they should be interpreted as upper-bound estimates of genetic control.

In this study, a shell color marker generated by varying feeds was used to record longitudinal shell-length data across ages. This method has also been reported in our previous experiments and in nutritional studies of several abalone species [30,31,32,33]. Heritability estimates for shell length varied across growth stages and showed a decreasing trend over time in the current study. However, in aquatic animals, the heritability of body size traits often changes across development and does not necessarily follow a monotonic pattern. For instance, in the Portuguese oyster (*Crassostrea angulata*) and the Pacific oyster (*Crassostrea gigas*), heritabilities for shell length and body weight exhibited stage-specific variation across larval, juvenile, and adult sampling periods, underscoring the importance of developmental stage in parameter estimation and the complexity of longitudinal datasets [34,35].

A key issue in longitudinal studies is the potential bias introduced when traits are recorded only for individuals that survive to harvest. Mortality is rarely random; weaker or slower-growing individuals are often more prone to die before data collection. If these individuals are systematically missing, phenotypic records of survivors may not fully represent the genetic variation in the original population [36]. This ‘selective genotyping’ can upwardly bias estimates of trait means and potentially downwardly bias estimates of genetic variance if the distribution is truncated. To rigorously address this in future evaluations, survival could be treated as a distinct binary trait and analyzed alongside growth in a multivariate model. This approach allows the model to account for the covariance between survival and growth, thereby correcting parameter estimates for the non-random missing data. Furthermore, this survivorship bias likely extends to meat yield estimates. Since meat yield was measured only at the final harvest on a subset of survivors, the data excludes individuals that succumbed to stress or poor conditions earlier in the cycle. If low meat yield is genetically correlated with lower survival (e.g., via general fitness or condition), our current estimates of meat yield heritability might be conservative due to reduced phenotypic variance in the surviving cohort. Nevertheless, the heritability estimates for shell length and total wet weight at harvest reported here remain consistent with published estimates for abalone species [8,37,38], suggesting they are likely adequate for initial breeding program design.

The phenotypic distribution of the foot color (Fc) trait deviated significantly from normality. Although the large sample size (n > 8000) theoretically allows Fc to be approximated as a continuous trait in a linear animal model, this approach yielded a lower heritability estimate (*h*^2^ = 0.26) than the cumulative multinomial threshold model (latent scale *h*^2^ = 0.46). This discrepancy is expected because threshold models are generally more appropriate for ordinal categorical traits and can provide less biased estimates of genetic variance components than linear models that treat ordinal scores as continuous [27,39]. Threshold models explicitly represent an underlying latent liability and accommodate non-normality on the observed scale, yielding estimates that are more consistent with the measurement scale of the trait [40,41]. The latent heritability of 0.46 indicates substantial genetic variation underlying Fc in this population, suggesting that selection against undesirable foot color scores could be effective. The moderate heritability of foot color suggests it can be effectively manipulated via breeding. This aligns with pigmentation studies in other aquatic invertebrates, such as the shell color of the Pacific oyster (*C. gigas*) and black-lipped pearl oyster (*Pinctada margaritifera*), where color traits often exhibit simple Mendelian inheritance or high heritability in threshold models [42,43]. Our results extend this knowledge to soft-tissue pigmentation in gastropods. Furthermore, regarding the genetic correlations, it should be noted that treating foot color as continuous in the multivariate analysis might slightly underestimate the true magnitude of correlations compared to a threshold–linear approach. However, given the non-significant and near-zero correlations observed between foot color and production traits (growth and meat yield), it is unlikely that model choice would alter the fundamental conclusion that foot color is genetically independent of productivity.

It is important to note that the sample size for meat traits (n = 680) was considerably smaller than for growth traits (n > 7900), which inherently impacts the statistical power of the analysis. The lower sample size may reduce the precision of these estimates compared to the growth traits. Consequently, the heritability estimate for meat yield reported here should be considered preliminary. Future studies with larger sacrificed cohorts or non-invasive imaging techniques would be beneficial to validate these findings and reduce the standard error. In contrast, the genetic evaluation of meat yield in 680 individuals did not produce the heritability magnitude reported in some previous studies of abalone and other aquatic species [44,45,46,47]. Apart from the severe imbalance that undermines the reliability and comparability of genetic parameter estimates for meat yield, several other factors may contribute to this discrepancy. First, meat yield is difficult to phenotype accurately. It is a ratio trait that may show limited phenotypic variation, and it requires slaughter, which constrains repeated measurements and can increase measurement noise [46,48,49]. In carcass and yield-related traits, improved phenotyping, including specialized or non-invasive techniques such as image-based methods, can enhance accuracy and, consequently, parameter estimation [48,50,51,52]. Second, environmental effects may be strong. Meat yield can be influenced by rearing conditions and seasonality; for instance, in the flat oyster (*Ostrea edulis*), condition index varies with temperature, nutrient availability, and chlorophyll-*a* [53]. Genotype-by-environment interactions may therefore affect trait expression and reduce consistency of genetic parameters across environments [54]. Third, sampling limitations may have reduced precision. Although 680 individuals are substantial, the estimates may still have large standard errors. In addition, if the sampled individuals were not fully representative of the population (e.g., due to selection bias or population structure), estimated genetic parameters may not be generalizable and may be biased [55].

A notable finding in this study was the contrast between the moderate heritability of meat weight (0.32 ± 0.05) and the low heritability of meat yield (0.14 ± 0.04). This discrepancy can be attributed to the statistical properties of ratio traits. Meat yield is a composite trait derived from meat weight divided by total wet weight. In ratio traits, measurement errors and environmental variability associated with both the numerator and the denominator accumulate, thereby inflating the residual variance relative to the genetic variance [49]. Biologically, this suggests that while the absolute growth potential of the abalone (total size and muscle mass) is under moderate genetic control, the physiological partitioning of resources between shell, viscera, and muscle is more heavily influenced by environmental factors or non-additive genetic effects. The lower heritability of meat yield compared to whole weight is not unique to this study. Similar trends have been observed in other aquaculture species; for example, in Atlantic salmon (*Salmo salar*), heritability estimates for fillet yield typically range from 0.11 to 0.24, which is notably lower than the heritability estimates for body weight, often found between 0.30 and 0.52 [56]. In the Portuguese oyster (*Crassostrea angulata*), genomic prediction for whole weight and meat yield (soft-tissue weight and condition index) indicated the potential for genomic information to improve selection efficiency for these environmentally sensitive traits [57].

Regarding selection strategies, the strong genetic correlation between total wet weight and meat weight (0.92) suggests that indirect selection for total weight is an efficient method to improve meat weight (total edible biomass). However, the genetic correlation between total wet weight and meat yield was low and non-significant. This indicates that selecting for faster growth will produce larger abalone with more meat in absolute terms, but it will not improve the meat-to-shell ratio. Consequently, if increasing the dressing percentage (yield) is a specific breeding objective, it cannot be achieved indirectly via growth selection and would require direct, likely genomic, selection approaches given the low heritability and phenotyping difficulty.

We found very low and non-significant genetic correlations between total wet weight and foot color. In some breeding programs, color traits are often used as indicators of growth due to positive genetic correlations reported in other aquaculture species or populations [17,58,59,60]. However, our results suggest that in *H. discus hannai*, selecting for increased total wet weight is unlikely to yield meaningful correlated improvements in foot color, and vice versa. These traits therefore appear largely under independent genetic control in this population, supporting the use of a multi-trait selection strategy if improvements in both growth and foot color are desired.

The independence of growth and quality traits (foot color and meat yield) necessitates a balanced breeding goal. Based on the limitations of this study, we propose several future directions. First, the low heritability of meat yield suggests that direct selection is challenging; larger sample sizes are needed in future studies to improve estimation precision. Second, to mitigate the potential observer bias inherent in commercial assessment routines, future research should adopt objective, non-invasive imaging techniques [61,62,63]. Finally, genomic selection (GS) offers a promising alternative for traits that are costly to phenotype or environmentally sensitive. Its success in other aquatic species [64,65,66,67] indicates a valuable path forward for abalone breeding programs.

## 5. Conclusions

This study evaluated the quantitative genetic basis of key traits in Pacific abalone (*Haliotis discus hannai*) to support quality-driven breeding. Analysis of 8001 individuals from 141 pedigreed families revealed moderate-to-high heritability for growth traits (shell length and total weight), indicating strong potential for genetic gain. Foot color and meat weight exhibited moderate heritability, whereas meat yield showed low heritability. Genetic correlations between total wet weight and foot color, as well as between total wet weight and meat yield, were low and non-significant. These results suggest that simultaneous improvement in growth and quality traits will require independent or multi-trait (genomic) selection approaches.

## Figures and Tables

**Table 1:** Descriptive statistics for traits of *H. discus hannai* population.

Trait (Units)	Age	N	Mean	SD	Min	Max
SL_6 (mm)	6 month	6259	23.85	2.68	10.95	41.64
SL_18 (mm)	18 month	5492	46.53	4.33	24.79	69.96
SL_24 (mm)	24 month	6843	62.64	4.46	42.9	80.68
SL_30 (mm)	30 month	7948	76.68	5.34	58.02	100.91
Tww (g)	30 month	7993	63.36	14.6	23.17	128.59
Fc (1–4)	30 month	7913	1.42	0.64	1	4
Mw (g)	30 month	680	30.64	13.03	11.66	66.33
My (%)	30 month	680	0.51	0.04	0.30	0.66

SL_6, SL_18, SL_24, SL_30, Tww, Fc, Mw, and My represent shell lengths at 6, 18, 24, 30 months, total wet weight, foot color, meat weight and meat yield. Max, maximum; Min, minimum; SD, standard deviation.

**Table 2:** Genetic correlations (above diagonal), phenotypic (below diagonal) correlations and heritabilities (in the diagonal) of the targeted traits in the *H. discus hannai* population.

	SL_6	SL_18	SL_24	SL_30	Tww
SL_6	0.71(0.06)	0.51(0.07)	0.31(0.09)	0.34(0.09)	0.31(0.09)
SL_18	0.38(0.03)	0.62(0.06)	0.31(0.09)	0.46(0.08)	0.38(0.08)
SL_24	0.30(0.03)	0.49(0.02)	0.45(0.05)	0.71(0.05)	0.53(0.07)
SL_30	0.24(0.03)	0.44(0.02)	0.63(0.01)	0.48(0.05)	0.90(0.02)
Tww	0.22(0.03)	0.37(0.02)	0.50(0.02)	0.82(0.01)	0.48(0.05)

SL_6, SL_18, SL_24, SL_30, and Tww represent shell lengths at 6, 18, 24, and 30 months, as well as total wet weight. Values in the parentheses are standard errors. All the data displayed above showed highly significant results (*p* < 0.05).

**Table 3:** Genetic correlations (above diagonal), phenotypic (below diagonal) correlations and heritabilities (in the diagonal) of the targeted traits in the *H. discus hannai* population from three bivariate models (i to iii).

i	Tww	Fc
Tww	0.48(0.05) **	−0.04(0.1)
Fc	−0.01(0.02)	0.26(0.03) *
ii	Tww	Mw
Tww	0.47(0.04) **	0.92(0.06) **
Mw	0.89(0.01) **	0.31(0.09) *
iii	Tww	My
Tww	0.48(0.05) **	−0.60(0.55)
My	−0.10(0.04)	0.14(0.24)

Tww, Fc, Mw, and My represent total wet weight, foot color, meat weight and meat yield. Values in the parentheses are standard errors. Significance: ** *p* < 0.01; * *p* < 0.05.

## Data Availability

The data that support the findings of this study are available from the corresponding author upon reasonable request.
